# Reduced transmission of *Klebsiella pneumoniae* carbapenemase-producing *K. pneumoniae* (KPC-KP) in patients with haematological malignancies hospitalized in an Italian hospital during the COVID-19 pandemic

**DOI:** 10.1093/jacamr/dlab167

**Published:** 2021-11-17

**Authors:** Alessandra Micozzi, Giovanni Manfredi Assanto, Laura Cesini, Clara Minotti, Claudio Cartoni, Saveria Capria, Giulia Ciotti, Danilo Alunni Fegatelli, Livia Donzelli, Maurizio Martelli, Giuseppe Gentile

**Affiliations:** 1 Department of Translational and Precision Medicine, Haematology, Sapienza University of Rome, Rome, Italy; 2 Department of Haematology, Oncology and Dermatology, Azienda Policlinico Umberto I, Rome, Italy; 3 Department of Public Health and Infectious Diseases, Sapienza University of Rome, Rome, Italy

## Abstract

**Objectives:**

During the lockdown that started in Italy on 10 March 2020 to address the COVID-19 pandemic, aggressive procedures were implemented to prevent SARS-CoV-2 transmission in SARS-CoV-2-negative patients with haematological malignancies. These efforts progressively reduced *Klebsiella pneumonia* carbapenemase-producing *K. pneumoniae* (KPC-KP) spread among these patients. Here we evaluated the potential effects of measures against COVID-19 that reduced KPC-KP transmission.

**Patients and methods:**

We analysed KPC-KP spread among 123 patients with haematological malignancies, hospitalized between March and August 2020, who were managed using measures against COVID-19. Their outcomes were compared with those of 80 patients hospitalized during the preceding 4 months (November 2019–February 2020).

**Results:**

During March–August 2020, 15.5% of hospitalized patients were KPC-KP positive, compared with 52.5% in November 2019–February 2020 (*P *<* *0.0001); 8% and 27.5% of patients in these two groups were newly KPC-KP positive, respectively (*P *=* *0.0003). There were eight new KPC-KP-positive patients during January 2020 and none during June 2020. The weekly rate of hospitalized KPC-KP-positive patients decreased from 50% during March 2020 to 17% during August 2020. Four KPC-KP bloodstream infections (BSIs) were experienced by 123 patients (3%) in March–August 2020, and seven BSIs (one fatal) by 80 patients (8%) in November 2019–February 2020 (*P *=* *0.02). Consumption and expense of ceftazidime/avibactam administered to KPC-KP-positive patients significantly decreased in March–August 2020.

**Conclusions:**

Aggressive strategies to prevent SARS-CoV-2 transmission were applied to all hospitalized patients, characterized by high levels of KPC-KP endemicity and nosocomial transmission. Such measures prevented SARS-CoV-2 infection acquisition and KPC-KP horizontal transmission. Reduced KPC-KP spread, fewer associated clinical complications and decreased ceftazidime/avibactam consumption represented unexpected ‘collateral benefits’ of strategies to prevent COVID-19.

## Introduction

We commenced applying measures to reduce the risk of SARS-CoV-2 transmission among patients admitted to the Department of Haematology, Policlinico Umberto I° Hospital, *Sapienza* University of Rome, Italy, starting at the end of January 2020. Our goal was to protect patients with haematological malignancies and healthcare personnel from contracting COVID-19^1^ as well as to guarantee continuity of care. From 10 March 2020 to the start of the lockdown because of the COVID-19 pandemic, our hospital was dedicated to the COVID-19 emergency. Consequently, planned activities were suspended, except for non-deferrable treatments, including those for patients with haematological malignancies.^2^

Starting in February 2020, we observed an unexpected and progressive decrease in the spread of *Klebsiella pneumoniae* carbapenemase-producing *K. pneumoniae* (KPC-KP) among patients undergoing treatment in the Department of Haematology. To evaluate a possible role of measures to counteract COVID-19 associated with the reduction of transmission KPC-KP, we compared the spread of KPC-KP colonization and infections among 123 patients hospitalized between March and August 2020. These patients were managed using measures against COVID-19, and their characteristics were compared with those of 80 patients hospitalized during the preceding 4 months (November 2019–February 2020).

## Patients and methods

We conducted a single-institution retrospective cohort study starting from the end of January 2020. We assessed fever, respiratory symptoms, ageusia, anosmia, previous contact with SARS-CoV-2-positive subjects and previous stays in high-risk COVID-19 areas among patients, healthcare personnel and visitors before they entered the Haematology Department.

From 10 March 2020, we performed procedures to prevent SARS-CoV-2 transmission associated with hospitalizations, conducted surveillance of inpatients according to national government guidelines and issued hospital directives to guide healthcare personnel.^1–3^ Hospitalization in the Department of Haematology is reserved for patients who tested negative in nasopharyngeal screens for SARS-CoV-2 RNA (real-time-PCR assay). Every effort was made to avoid hospitalization of SARS-CoV-2-positive patients, including nasopharyngeal screening performed the day prior to entry, upon entry (negative patients entered the ward after an appropriate triage to rule out fever or respiratory signs and symptoms and the patient stayed in an isolation area while waiting for the virology report) and weekly during hospitalization. Immediate transfer of SARS-CoV-2-positive patients to COVID-19 wards was mandatory.^2^

Personal protective equipment (PPE) recommended for COVID-19 (gloves, gown, face-masks) was always utilized by healthcare personnel in contact with all hospitalized patients regardless of COVID-19-test status, with particular emphasis on using an alcohol-based sanitizer to disinfect the hands. Healthcare personnel were trained on proper wear, removal and disposal of PPE. External contact was reduced, visits were restricted (one SARS-CoV-2-negative visitor for 1 h each day) and patients and visitors were requested to remain isolated in their rooms. We limited, to the extent possible, patient admissions or temporary transfers for the purpose of consulting or diagnostics from and to other wards where they might acquire SARS-CoV-2 infection.

Starting in 2012, we performed active surveillance to detect the spread of KPC-KP and applied control measures.^4^ Screening for KPC-KP rectal colonization (real-time-PCR assay) was performed upon admission and weekly during hospitalization; KPC-KP carriers were assigned to isolation rooms or to double-occupancy rooms. We monitored KPC-KP carriers and KPC-KP bloodstream infections (BSIs). The combination of ceftazidime/avibactam, tigecycline and gentamicin was used as standard treatment for confirmed KPC-KP infections^5^ and preemptively for KPC-KP carriers, for the purpose of empirical treatment of febrile neutropenia.^4^^,^^6^^,^^7^ Ceftazidime/avibactam treatment was strictly reserved for the indications described above.

We recorded patients’ demographics, underlying disease, number of hospitalizations, febrile neutropenia episodes per patient and KPC-KP BSI and its associated mortality during the two periods. According to KPC-KP colonization or infection, we recorded the number of KPC-KP-positive patients (new carriers versus known positives upon admission) and KPC-KP-negative patients hospitalized during the two periods. We presumed that KPC-KP was horizontally transmitted among KPC-KP-negative patients upon admission, during a previous hospitalization or both. The daily number of hospital beds occupied by KPC-KP-positive and KPC-KP-negative patients was calculated and presented as weekly and monthly data for the two study periods. We collected data related to the administration of ceftazidime/avibactam (days of treatment [DOT], consumption and cost).

Data related to the subject population’s characteristics are presented as the mean and standard deviation, frequencies or percentages. Bayesian Change Point (BCP) analysis^8^ was implemented to identify rapid shifts in time-series data. One-way analysis of variance (ANOVA) was applied to determine whether the mean values were equivalent during each study period. The Durbin–Watson and Shapiro–Wilk tests were performed to assess autocorrelation and normality, respectively, of the residuals obtained using the ANOVA model. The analyses were performed using R (version 3.6.2) and the BCP package 4.0.3 was used to perform the BCP analysis.

### Ethics

The research was conducted in accordance with the Declaration of Helsinki and national and institutional standards. The Internal Review Board of the Haematology Department approved the study.

## Results

From March to August 2020, no patient tested positive for SARS-CoV-2 during hospitalization. The mortality rates of patients hospitalized during the two study periods were similar, and the majority of patients with acute leukaemia underwent intensive chemotherapy. Episodes of febrile neutropenia were experienced by a high proportion of patients during both study periods, and their mortality rates were comparable (Table 1). The overall rates of KPC-KP-positive patients hospitalized and newly KPC-KP-positive patients were significantly lower in March–August 2020 compared with November 2019–February 2020 (15.5% versus 52.5% [*P *<* *0.0001] and 8% versus 27.5% [*P *=* *0.0003], respectively) (Table 1).

**Table 1.** Patients' characteristics and transmission of KPC-KP

	November 2019–February 2020	March 2020–August 2020	*P* ^a^
Hospitalized patients, *n*	80	123	
Age, years, median (range)	56 (23–81)	60 (22–80)	0.08
Male, *n* (%)	39 (49)	68 (55)	0.3
Underlying disease, *n* (%)			
Acute leukaemia	35 (48)	46 (38)	0.3
Lymphoma	27 (30)	48 (39)	
Multiple myeloma	12 (15)	14 (11)	
Other	6 (7)	15 (12)	
Reason for hospitalization, *n* (%)			
High-dose chemotherapy	36 (45)	52 (43)	0.2
Autologous SCT	20 (25)	24 (21)	
Immunotherapy	6 (8)	12 (8)	
Other	15 (22)	35 (28)	
Patients previously submitted to allogeneic SCT, *n* (%)	6 (7)	7 (6)	0.6
Hospitalizations per patient, *n* (range)	2 (1–5)	2 (1–6)	
Total KPC-KP-positive patients, *n* (%)	42 (52.5)	19 (15.5)	<0.001
New KPC-KP-positive patients	22 (27.5)	10 (8)	0.0003
Patients known to be KPC-KP positive at hospitalization	20 (25)	9 (7.5)	
Patients who developed febrile neutropenia during hospitalization, *n* (%)	58 (72)	80 (65)	0.2
KPC-KP bloodstream infections, *n* (%)	7 (8)	4 (3)	0.02
KPC-KP bloodstream infections developed in KPC-KP carriers, *n*/*N* (%)	7/42 (17)	4/19 (21)	0.7
Deaths for any reason, *n* (%)	9 (11)	13 (10.5)	0.5
KPC-KP-related death	1 (1)	0	
Ceftazidime/avibactam			
Total consumption, vials	940	740	
Median consumption/month, vials	245	110	0.03
Total DOT	313	247	
Mean DOT/month	81	36	0.002
Median cost/month, €	19 550	10 000	0.03

SCT, stem cell transplant.

a Median values of the two periods were compared using the Mann–Whitney test.

**Figure 1.**
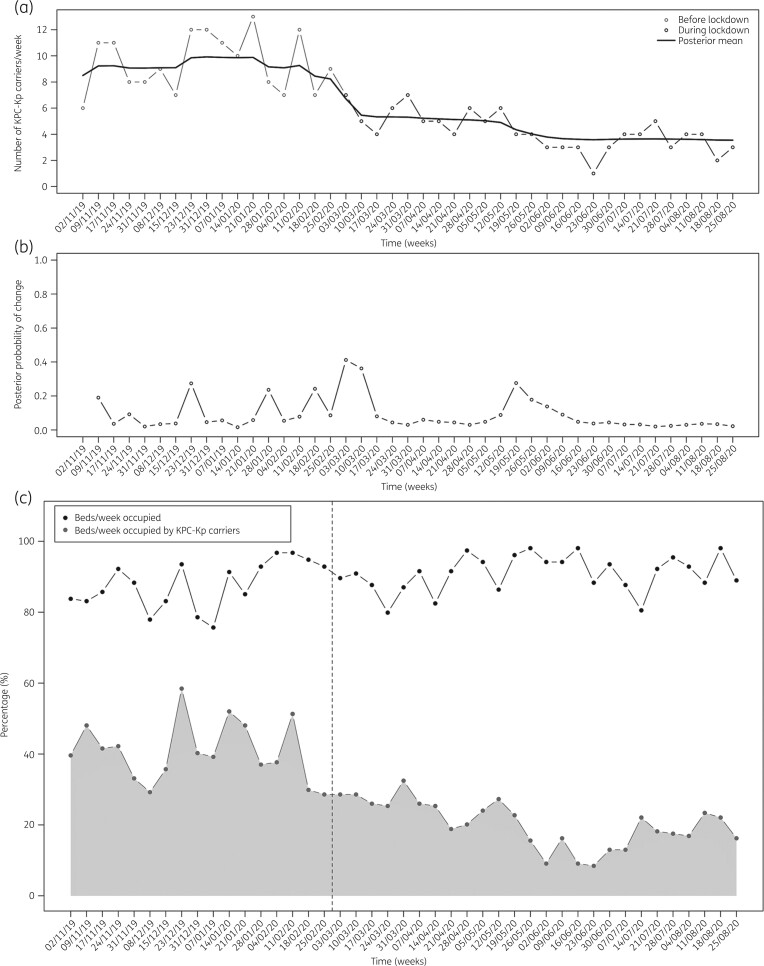
(a) Posterior means of the number of KPC-KP carriers hospitalized each week from November 2019 to August 2020. (b) Corresponding posterior probability of a change at each timepoint (week). (c) Percentage of beds per week occupied (black dotted line) and percentage of beds per week occupied by KPC-KP carriers (grey dotted line) from November 2019 to August 2020. The dashed vertical line indicates the start of the COVID-19-related lockdown in Italy.

During the November 2019–February 2020 study period, the median weekly rates of hospitalized KPC-KP-positive patients ranged from 30% to 50%. From March 2020, the rate progressively decreased, reaching its nadir (17%) during August 2020. The number of new KPC-KP carriers identified per month decreased from eight during January 2020 to zero during June 2020 (Figure 1).

The number of hospitalizations did not decrease during the March–August 2020 study period. During both periods, the ward capacity of 23 hospital beds was 86% (median; range 81%–89%). The rates of beds occupied by KPC-KP carriers progressively decreased from 51% during January 2020 to approximately 20% during August 2020 (Figure 1).

Seven KPC-KP BSIs (one fatal) developed among the 80 patients (8%) hospitalized during the November 2019–February 2020 study period and among 4 of 123 patients (3%) during the March–August 2020 study period (*P *=* *0.02). The rates of KPC-KP carriers who developed KPC-KP BSIs were similar during the two study periods (17% and 21% during November 2019–February 2020 and March–August 2020, respectively) (Table 1).

The DOT, monthly consumption and expense for ceftazidime/avibactam treatment significantly decreased during the March–August 2020 study period (Table 1). Ceftazidime/avibactam consumption progressively decreased from 245 vials/month (mean; range 90–320) during the November 2019–February 2020 study period to 110 vials/month (mean; range 90–320) during the March–August 2020 study period (*P *=* *0.03). The DOT decreased from 81 DOT/month to 36 DOT/month in the November 2019–February 2020 and March–August 2020 study periods, respectively (*P *=* *0.002). The expense associated with the administration of ceftazidime/avibactam progressively decreased from €26 000 during January 2020 to €8200 during August 2020 (range €7300–26 050; median costs/month were €19 550 and €10 000 during the November 2019–February 2020 and March–August 2020 study periods, respectively [*P *=* *0.03]) (Table 1).

## Discussion

During the COVID-19 pandemic, we documented a significant reduction in nosocomial transmission of KPC-KP, which differs from the results of other studies^9^ reporting increases in the rates of infection and transmission of antimicrobial-resistant organisms (MDROs), mainly in the ICU setting.^10^ These events are related to overcrowding of hospitals, high workloads leading to increased non-compliance with contact precautions, increased administration of antimicrobials, interruptions in continuity of antimicrobial stewardship programmes and a high intensity of care. In Italy, KPC-KP has established high-level endemicity.^11^ Despite the application of control measures, nosocomial transmission of KPC-KP and the prevalence of colonized hospitalized patients are high, particularly in haematological wards.^4^^,^^7^ Rectal colonization by KPC-KP represents a risk factor for KPC-KP BSI, particularly in neutropenic patients.^4^^,^^7–15^ Moreover, KPC-KP-BSI-related mortality in this population is very high if initial aggressive treatment is not administered.^4^^,^^6^^,^^7^^,^^13–15^

Our historical data show a progressive increase in the prevalence of KPC-KP starting in 2012. During 2018, 43% of our hospitalized patients were carriers of KPC-KP (26% new carriers), and the days of hospitalization of KPC-KP-positive patients were 2946 of 9354 (31.5% of the total) (A. Micozzi, L. Cesini and C. Minotti, unpublished data). These values remained substantially unchanged, and during the 4 months preceding March 2020, approximately 50% of our hospitalized patients carried KPC-KP. After March 2020, the percentage of KPC-KP-positive patients progressively decreased, reaching the lowest rate during August 2020.

Measures such as hand hygiene, social distancing and self-isolation to prevent SARS-CoV-2 infection and transmission in our haematology ward might have indirectly supplemented the procedures to prevent the transmission of MDROs.^16^ Increased contact precautions were implemented to prevent the risk of airborne transmission SARS-CoV-2 through microdroplets, reduce direct contact and prevent faecal-oral transmission modes. These measures, which were practised by healthcare personal during the COVID-19 pandemic, were extended to all hospitalized patients, regardless of KPC-KP colonization. Such efforts reduced the spread of KPC-KP. Moreover, the fear of infection with SARS-CoV-2, transmission to patients and family members, or both, particularly during the lockdown, may have strengthened adherence to these procedures.

Fewer concomitantly hospitalized KPC-KP carriers contributed to the reduction of transmission of KPC-KP. The large number of carriers subjected to contact precautions increases the risk of isolation precaution transgressions, thus favouring the spread of KPC-KP,^4^^,^^7^ which is further facilitated by frequent clinical emergencies experienced by patients with haematological malignancies who undergo intensive chemotherapy.^4^ Further, intensive treatment of haematological malignancies, particularly acute leukaemia, includes repeated courses of chemotherapy administered during multiple hospitalizations. Thus, the acquisition of KPC-KP, particularly during the initial phase of treatment, contributes to the burden of KPC-KP in the ward during present and subsequent hospitalizations.

Colonization of the rectum by KPC-KP is a risk factor for KPC-KP BSI.^4^^,^^7–14^ We show here that the rates of carriers who developed KPC-KP BSIs were similar during the two study periods. However, the reduced number of KPC-KP carriers hospitalized during the March–August 2020 study period may explain the observed overall decrease in the frequency of KPC-KP BSIs. Moreover, despite episodes of febrile neutropenia that similarly occurred during the two study periods, we found a significant reduction in the consumption of ceftazidime/avibactam, which is mainly preemptively administered to KPC-KP carriers as an empirical treatment of febrile neutropenia.^7^

### Conclusions

We applied aggressive strategies that successfully prevented the transmission of SARS-CoV-2 to all hospitalized patients, particularly those undergoing chemotherapy. Moreover, such measures prevented the horizontal transmission of KPC-KP. This unexpected ‘collateral benefit’ reduced the spread of KPC-KP, reduced KPC-KP-associated clinical complications and reduced ceftazidime/avibactam consumption. These benefits persisted after the end of the study period, simultaneously with the continued application of prevention strategies against SARS-CoV-2. In high-level endemicity countries, these results support the conclusion that aggressive prevention strategies to prevent the transmission of MDROs should be applied to all hospitalized patients, regardless of the condition of a carrier of an MDRO.

## Funding

This study was carried out as part of our routine work.

## Transparency declarations

None to declare.

### Author contributions

A.M.: conception and design of the study, acquisition of data, analysis and interpretation of data and preparation of the manuscript. G.M.A.: acquisition of data, analysis and interpretation of data and preparation of the manuscript. L.C.: acquisition of data, analysis and interpretation of data and preparation of the manuscript. C.M.: acquisition of data, analysis and interpretation of data and review of the manuscript. C.C.: interpretation of data and review of the manuscript. S.C.: interpretation of data and review of the manuscript. G.C.: acquisition and analysis of data. D.A.F.: performing statistical analysis. L.D.: acquisition and analysis of data. M.M.: review of the manuscript. G.G.: preparation of the manuscript.
